# How to Harness the Power of GPT for Scientific Research: A Comprehensive Review of Methodologies, Applications, and Ethical Considerations

**DOI:** 10.1007/s13139-024-00876-z

**Published:** 2024-08-12

**Authors:** Ki-Seong Park, Hongyoon Choi

**Affiliations:** 1 Department of Nuclear Medicine, Chonnam National University Hospital, 42 Jaebong-ro, Dong-gu, Gwangju, 501-757 Republic of Korea; 2 Department of Nuclear Medicine, Seoul National University Hospital, 101 Daehak-ro, Jongno- Gu, Seoul, 03080 Republic of Korea; 3 Department of Nuclear Medicine, Seoul National University College of Medicine, 101 Daehak- ro, Seoul, 03080 Jongno-Gu Republic of Korea

**Keywords:** Generative Pre-trained Transformer (GPT), Natural Language Processing, Research Efficiency, Prompt Engineering, Interdisciplinary Collaboration

## Abstract

The rapid advancements in natural language processing, particularly with the development of Generative Pre-trained Transformer (GPT) models, have opened up new avenues for researchers across various domains. This review article explores the potential of GPT as a research tool, focusing on the core functionalities, key features, and real-world applications of the GPT-4 model. We delve into the concept of prompt engineering, a crucial technique for effectively utilizing GPT, and provide guidelines for designing optimal prompts. Through case studies, we demonstrate how GPT can be applied at various stages of the research process, including literature review, data analysis, and manuscript preparation. The utilization of GPT is expected to enhance research efficiency, stimulate creative thinking, facilitate interdisciplinary collaboration, and increase the impact of research findings. However, it is essential to view GPT as a complementary tool rather than a substitute for human expertise, keeping in mind its limitations and ethical considerations. As GPT continues to evolve, researchers must develop a deep understanding of this technology and leverage its potential to advance their research endeavors while being mindful of its implications.

## Introduction

The field of natural language processing (NLP) has witnessed remarkable advancements in recent years, particularly with the development of large language models such as the Generative Pre-trained Transformer (GPT) by OpenAI [1]. GPT has revolutionized the way researchers interact with and utilize artificial intelligence in various domains, including scientific research. By leveraging vast amounts of text data for pre-training, GPT has gained the ability to understand and generate human-like language with impressive accuracy and fluency [2]. This versatility has opened up new possibilities for researchers, allowing them to harness GPT’s capabilities to enhance their research processes and uncover novel insights.

The potential applications of GPT as a research tool are extensive and promising. From literature review and data analysis to hypothesis generation and manuscript writing, GPT can assist researchers at every stage of the research process [3]. However, to fully utilize the power of GPT, researchers need to understand its strengths, limitations, and the art of prompt engineering—crafting effective prompts to guide GPT towards desired outcomes. This review article aims to provide a comprehensive overview of GPT and its potential applications in scientific research. We will explore the core functions of GPT, discuss the evolution of GPT models, and introduce the concept of prompt engineering. Through real-world case studies and examples, we will demonstrate how researchers can successfully apply GPT to accelerate scientific discovery while also addressing the limitations and ethical considerations surrounding its use.

### Core Functions of GPT

GPT’s core functions can be categorized into three main areas: Natural Language Understanding (NLU), Natural Language Generation (NLG), and emergent abilities [4]. NLU refers to GPT’s ability to comprehend the meaning of text at a deep level, going beyond the surface-level meaning of words and considering the context, logical relationships between sentences, and the overall theme and tone of the text [5]. This enables GPT to grasp the true intent behind a user’s question or request, making it a valuable tool for tasks such as literature review and data analysis. Notably, compared to previous deep learning models for NLP, NLU benefits greatly from the Transformer model [6], which uses an attention mechanism to process large chunks of text simultaneously. This allows the model to handle long sequences effectively, capturing dependencies between distant parts of the text. Consequently, Transformers excel in understanding context and maintaining long-term memory in language, enhancing their performance in various tasks.

NLG, on the other hand, pertains to GPT’s capability to generate grammatically correct and semantically coherent sentences based on its understanding of the input [5]. Instead of merely combining pre-existing sentences from a dictionary, GPT selects appropriate words based on the context and creates innovative expressions, resembling the way humans write. This feature is particularly useful for tasks such as manuscript preparation and report writing [7].

In addition to NLU and NLG, GPT possesses emergent abilities that were not explicitly taught during the pre-training process. These abilities include instruction following, in-context learning, and step-by-step reasoning [4]. Instruction following refers to GPT’s ability to understand and execute simple instructions, enabling researchers to direct GPT towards specific tasks. In-context learning allows GPT to learn new concepts from a few examples, demonstrating its adaptability and quick learning capabilities. Step-by-step reasoning enables GPT to solve problems through a series of logical steps, making it suitable for complex research tasks that require sequential thinking.

The synergistic effects of GPT’s core functions and emergent abilities make it a powerful tool for researchers. By combining NLU, NLG, and emergent abilities, GPT can assist researchers in various stages of their work, from literature review and hypothesis generation to data analysis and manuscript writing [8]. However, to effectively leverage GPT’s capabilities, researchers must understand its strengths and limitations and learn to combine their own creativity and domain expertise with GPT’s generative power.

### Evolution and Key Features of GPT Models

The landscape of GPT models is rapidly evolving, with each new version offering enhancements and capabilities. GPT-3.5, the model powering the popular ChatGPT, has gained significant attention due to its ability to engage in natural conversations with users and free accessibility [9]. However, the more advanced GPT-4 model has taken GPT’s capabilities to new heights. One of the most significant features of GPT-4 is its ability to process multimodal inputs, understanding and analyzing not only text but also images, audio, and video data [10]. This feature is particularly valuable for researchers working with diverse data types, as it enables them to extract insights from multiple sources simultaneously. Moreover, GPT-4 boasts enhanced analysis capabilities, allowing it to independently write programming code to analyze given data, mostly in the form of Excel files [11]. This feature streamlines the data analysis process, saving researchers time and effort in writing complex code for data manipulation and visualization.

Another notable feature of GPT-4 is the ability to create customized GPT, sometimes called GPTs, for domain-specific tasks. Researchers can create GPTs by pre-defining prompts suitable for a specific task, uploading necessary materials, and using them whenever needed [12]. This approach reduces repetitive work and allows for continuous performance improvement through consistent management. For example, medical researchers can create a medical-specific GPTs by uploading medical papers or guidelines, enabling the GPTs to refer to the uploaded files when answering incoming questions.

When designing GPTs, researchers should systematically define the model’s role, instructions, knowledge, capabilities, and actions [13]. Instructions guide the tasks GPTs should perform, while knowledge provides the necessary background information for task completion. Capabilities specify the functional elements that GPTs should possess, such as web searches, drawing pictures, or conducting data analysis. Actions enable the connection with other APIs using the OpenAPI format [14], allowing GPTs to utilize external services. For instance, by using the Entrez API provided by the National Center for Biotechnology Information (NCBI) [15], ChatGPT can search PubMed and find relevant literature on a given topic.

GPTs can be shared with desired individuals through links, and if made public on the GPT Store, they can be accessed and used by a wide range of people [16]. Conversely, researchers can search for and use desired GPTs from the store, such as the “Consensus” [17], which contains a literature database and is frequently used for literature searches in ChatGPT.

For researchers who wish to combine various functions and automate them to suit their needs, the GPT API offers a powerful solution. Packages like LangChain allow for the integration of diverse functions, databases, and artificial intelligence models, enabling flexible designs [18]. However, using the GPT API requires coding skills and incurs costs. Nonetheless, we are able to use the GPT API to process large amounts of data, for example, to process large unstructured text reports and turn them into structured information, we no longer design a rule-based algorithm, but instead design an NLP-based algorithm that uses the GPT API. Additionally, performance variations still occur based on the prompts used, necessitating the prompt engineering techniques described in the next section.

A new version of GPT-4, called GPT-4o [19], has been released with significantly enhanced multimodal input processing and output generation capabilities. This model can integrate and process text, speech, and images within a single model, enabling highly natural conversations in real-time interactions with users. It particularly shines when used through smartphones, where users can receive more intuitive and efficient assistance via voice commands and image inputs. For instance, if a user shows the inside of their refrigerator using their smartphone camera and asks for recipe suggestions via voice, GPT-4o can recommend possible dishes and provide recipes through both voice and text.

### Prompt Engineering

Designing Effective Prompts for GPT Prompt engineering is a crucial skill for effective GPT utilization, as it involves providing optimal inputs to GPT to elicit desired outputs [20]. The performance of GPT can vary significantly depending on the prompt design, even when using the same model, underscoring the importance of mastering prompt engineering.

**Fig. 1:**
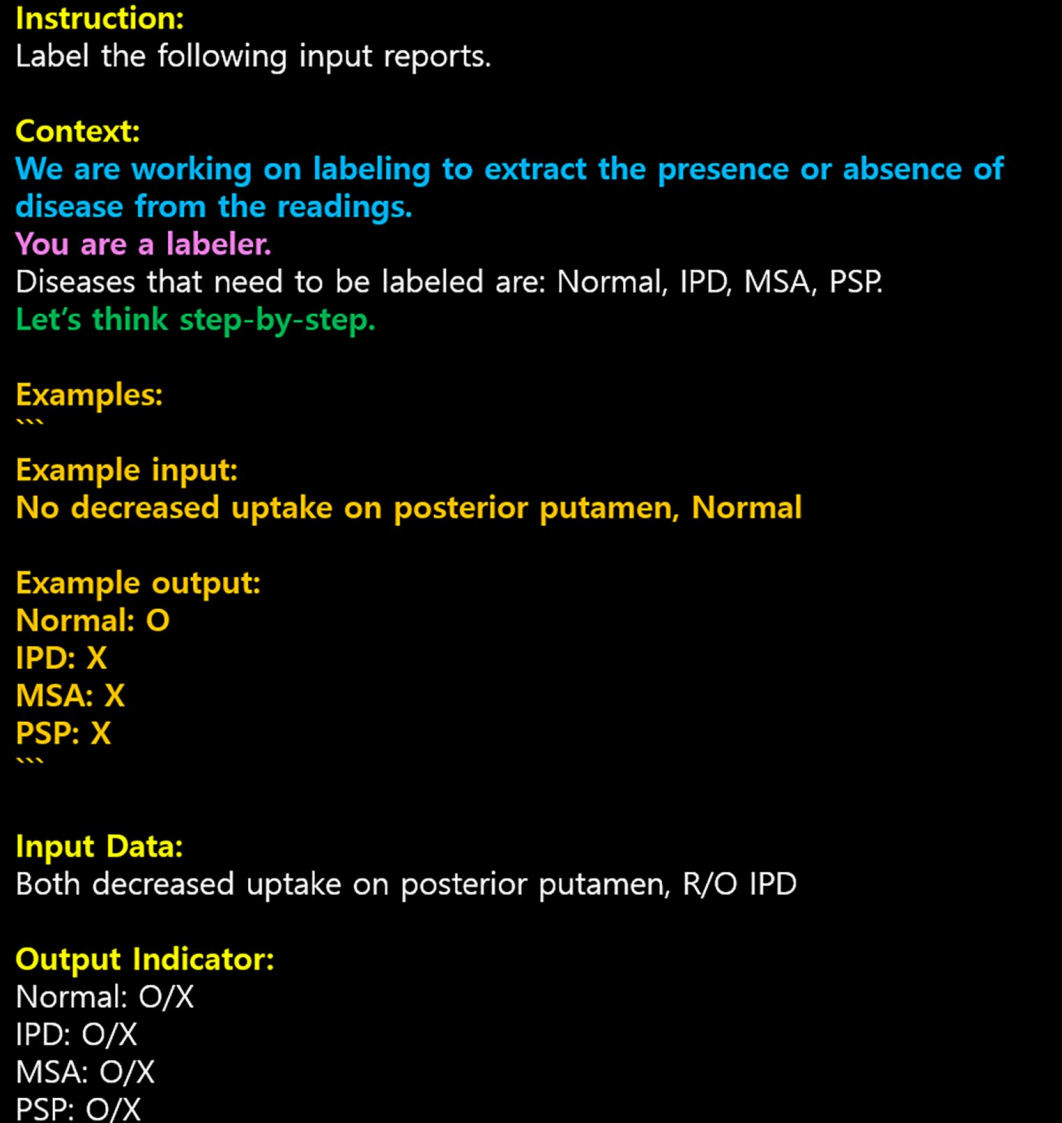
Example of a prompt format. Yellow represents the Prompt Structure, blue indicates the parts that need to be written with Context and Clarity, purple denotes the section where a role is assigned to GPT, green represents the part utilizing Chain-of-Thought, and orange signifies the portion where examples are provided to help GPT understand the task

While prompts can have various structures, a basic format consisting of instruction, context, input data, and output indicator is recommended (Fig. 1). The instruction clearly specifies the task GPT should perform, with concise and specific instructions enhancing GPT’s understanding. The context provides background information necessary for task completion, helping GPT understand the context. Input data is the actual input GPT needs to process, and its format and content should be clearly defined. The output indicator designates the format of the output GPT should generate, ensuring structured and consistent results.

To create effective prompts, researchers should follow three key principles: clarity and specificity, providing roles and context, and inducing step-by-step reasoning [21]. Prompts should be as clear and specific as possible, avoiding ambiguous or abstract expressions and using simple and direct language. If necessary, examples can be used to explain the task.

Providing roles and context is essential for GPT to generate appropriate language. Specifying GPT’s role (e.g., research assistant, data analyst) and the background of the task in the prompt can help GPT understand its purpose and the context of the conversation. Additionally, providing relevant examples, a technique known as “few-shot learning,” can further enhance GPT’s understanding.

For complex tasks, inducing step-by-step reasoning is an effective approach. By specifying intermediate steps or requiring step-by-step output in the prompt, GPT can be encouraged to think sequentially [22]. This helps break down complex problems into manageable subtasks, enabling GPT to provide more accurate and relevant responses. To apply these principles to prompts, researchers should have a clear understanding of the task’s purpose and procedure. The task should be broken down into subtasks, and the requirements for GPT at each step should be defined.

Alternatively, another promising technology is Zero-shot Chain-of-Thought (CoT) prompting, which encourages the model to generate a series of intermediate reasoning steps before providing the final answer, even without explicit examples in the prompt. This can be achieved by simply adding a phrase like “Let’s think step by step” to the prompt [23]. Zero-shot CoT has been shown to improve the performance of language models on a variety of complex reasoning tasks, demonstrating the effectiveness of eliciting step-by-step thinking in GPT.

Prompt engineering is a critical skill for GPT utilization, but users often face a steep learning curve when mastering this new skill. Many users, initially frustrated by ChatGPT’s seeming inability to understand their intentions, abandon the tool prematurely. However, crafting effective prompts requires dedicating time and effort to understand the principles of prompt engineering and iteratively refining prompts to guide the model towards desired outputs. This process can be likened to teaching rules to a four-year-old child, demanding patience and persistence in repeating instructions. Similarly, positive reinforcement is crucial; research has shown that praising GPT for its outputs can lead to improved results [24]. By providing encouragement for correct behaviors and constructive feedback for misaligned responses, users can help GPT progressively align its outputs with their expectations.

Another useful technique is Retrieval-Augmented Generation (RAG), which improves truthfulness and helps GPT models integrate the latest research findings by retrieving relevant information from external databases or documents and using it to generate responses [25]. RAG consists of two steps. First, it understands the request and searches for the necessary information. Then, the GPT model generates a more accurate and up-to-date answer based on the retrieved information. RAG is particularly useful in rapidly evolving fields like medicine and science, where it can help researchers quickly summarize the latest treatment methods for a specific disease, saving time and increasing research efficiency. When used in conjunction with prompt engineering, RAG can significantly enhance the accuracy and efficiency of research utilizing GPT models.

### Applications of GPT in Scientific Research

The potential applications of GPT in scientific research are vast and diverse. In this section, we present use cases highlighting how GPT can be utilized in different stages of the research process, including manuscript preparation, research data analysis, and research trend analysis.

### Literature Review

GPT can be used to search and review literature. GPT-4 has its own search functionality, and you can also use custom GPTs for literature search, such as the previously mentioned “Consensus” from the GPT Store [17], or utilize APIs to access other literature search services. One advantage of using ChatGPT for literature search is the ability to query using natural language. In the case of Pubmed or Google Scholar, searches often cannot be performed using natural language, and controlled vocabularies such as MeSH terms must be used, requiring the creation of sophisticated search queries. In contrast, with GPT, even if you query in natural language, it can be converted into controlled vocabularies for searching, thereby enhancing user convenience. Additionally, based on the content of the retrieved literature, you can receive summaries or engage in question-answering, making the workflow more streamlined.

However, there are limitations. In cases where GPT provides an answer without conducting a search, it occasionally provides information about non-existent literature. Moreover, it may not be able to recommend a large number of papers at once and may have a limited search scope, unable to find literature not included in the connected databases. At times, it may be difficult for GPT to find studies as relevant as those carefully selected by researchers directly reviewing Pubmed or Google Scholar.

### Research Data Analysis

GPT can also be leveraged for research data analysis, particularly in handling large volumes of unstructured data. In the medical research domain, for example, researchers often deal with vast amounts of medical report data. Extracting specific disease names or medical terms from this textual data can be a daunting task, but GPT can perform it remarkably well [26].

**Fig. 2:**
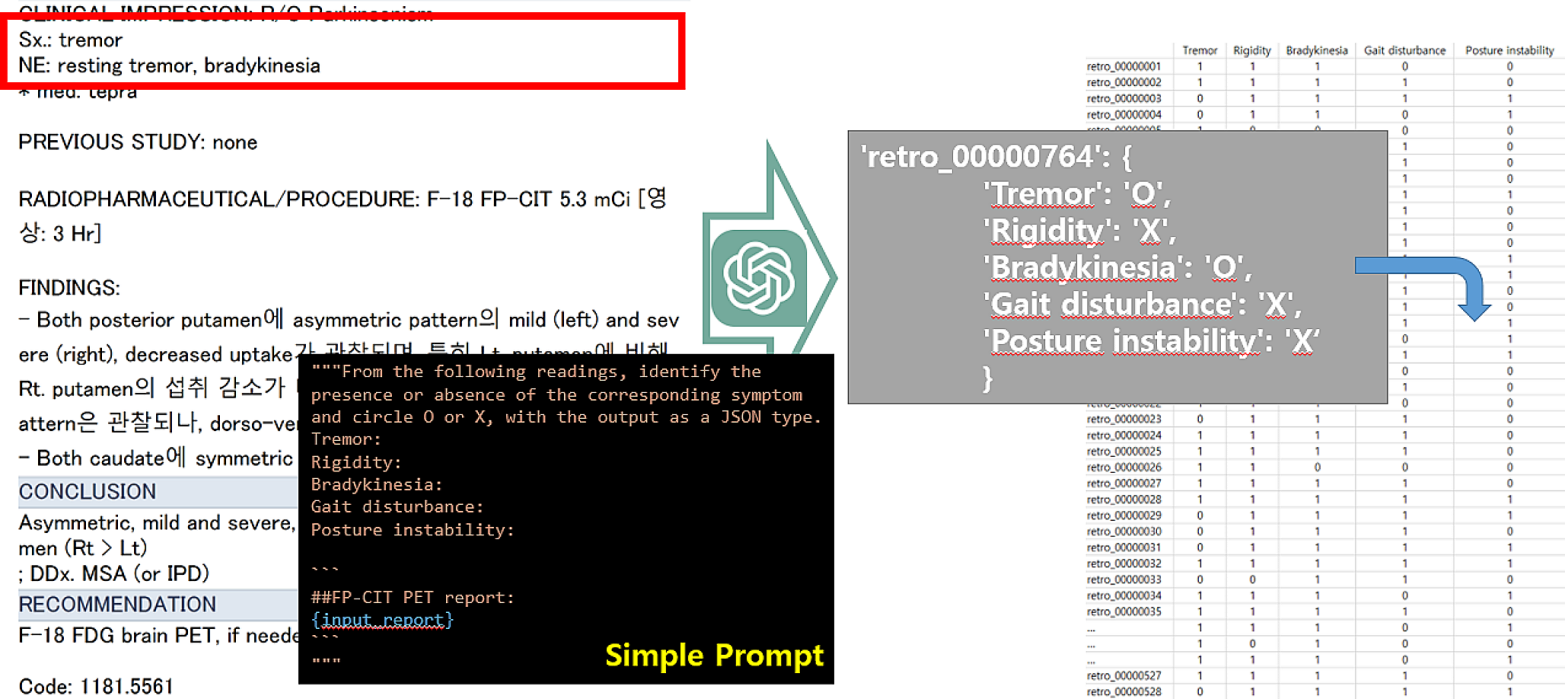
An example of using GPT to convert unstructured data into structured data through labeling. The information recorded about the Neurological Examination in the radiology report is being transformed into a table format Excel file via GPT

By providing appropriate prompts, researchers can request GPT to analyze the report contents and extract the desired information. Furthermore, GPT can be used to convert unstructured data into a structured format, enabling researchers to utilize the data for statistical analysis (Fig. 2). This process of data structuring and cleaning can significantly reduce the time and effort required for manual data preprocessing.

Moreover, GPT has the ability to write and execute analysis code on its own. Researchers can upload data files and have GPT read, analyze, and derive results independently. However, even in this case, as the prompts become longer and more complex, there may be instances where GPT fails to understand the instructions. Therefore, a step-by-step approach is recommended. When used in this manner, the calculations are performed based on the generated analysis code rather than within the GPT model itself, resulting in relatively accurate outcomes. However, verification is still crucial. Additionally, reviewing the analysis code for potential errors requires basic coding skills.

In addition to data analysis, GPT can assist researchers in various stages of manuscript preparation [27–29]. While GPT can help organize ideas and provide suggestions based on key elements like the title, abstract, figures, and tables, it is crucial that authors themselves craft the core content and ensure the logical coherence and accuracy of the generated text. After receiving assistance from GPT, researchers must thoroughly review the generated content, verify facts, and make necessary revisions. GPT can also help modify the reference style according to the target journal’s guidelines or assist in translating the manuscript. However, providing GPT with the entire author guidelines may not yield optimal results, and it is recommended to use only the relevant sections when seeking GPT’s assistance.

GPT is a valuable tool for research data analysis and manuscript preparation, but should not replace human expertise and critical thinking. Journals, including the Lancet, recommend that the use of AI such as GPT be used only to improve the readability and language of papers. Additionally, the majority of publishers and journals do not recognize generative AI as authors, and require disclosure of how and for what purpose generative AI was used [30]. Therefore, researchers must take responsibility for the final content and ensure that their manuscripts meet the highest standards of academic integrity.

### Research Trend Analysis

**Fig. 3:**
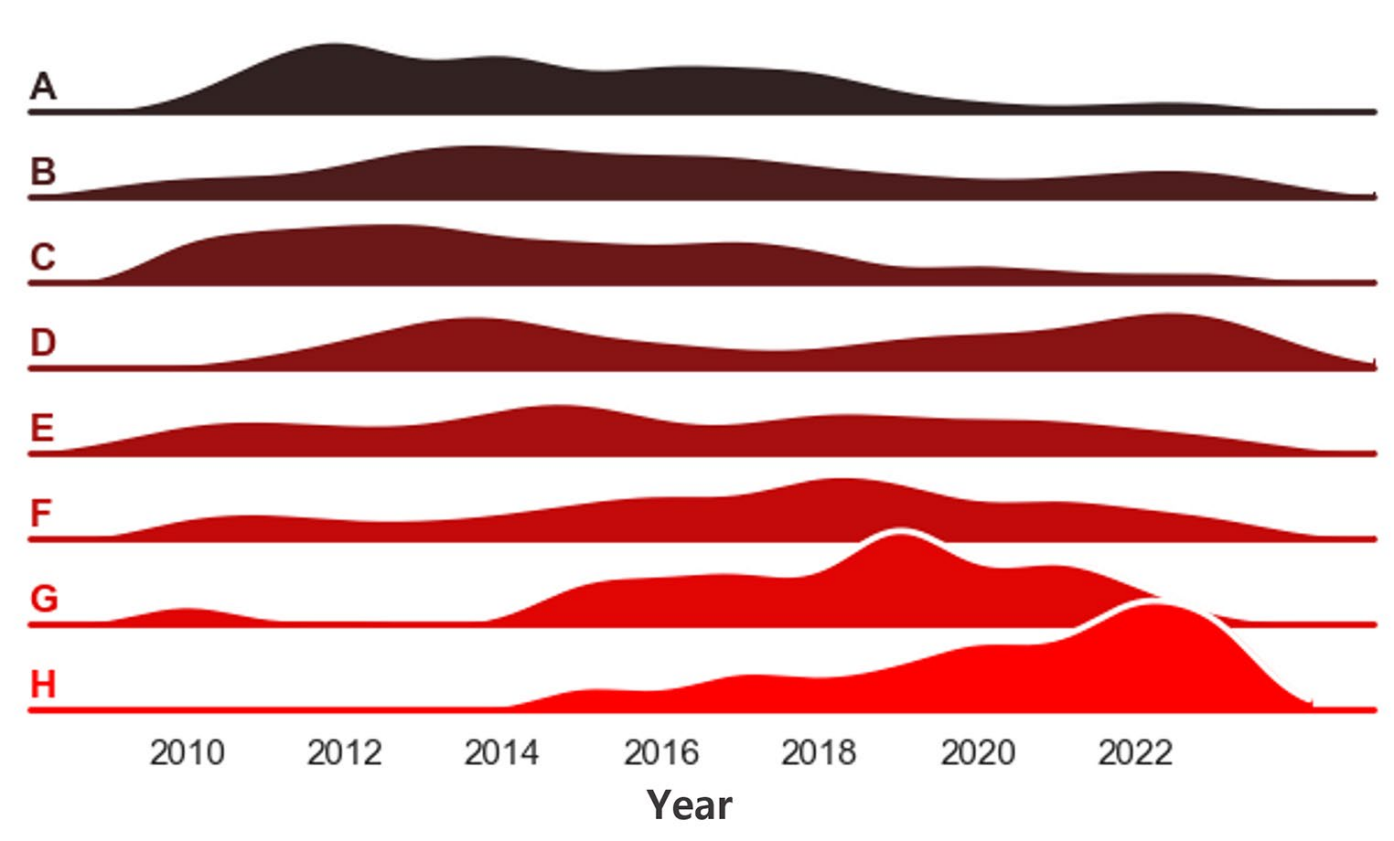
Research trend analysis of the Nuclear Medicine and Molecular Imaging journal from 2010 to 2023 using GPT. **A**: Explores the evaluation of metabolic response to chemotherapy using FDG PET/CT and the utility of metabolic volumetric indices for staging and prognosis prediction, **B**: Discusses the role of nuclear medicine in cardiovascular diseases, including myocardial damage assessment, treatment efficacy evaluation, and functional assessment of coronary artery disease. **C**: Presents the potential applications of PET/CT in various diseases, including bone metastasis assessment (e.g., breast cancer, liver cancer), differential diagnosis (e.g., CNS lymphoma vs. infection), and IgG4-related diseases. **D**: Focuses on the use of PET imaging for differential diagnosis of neurological disorders like Parkinson’s disease, quantitative analysis methods for PET, and factors influencing PET study results. **E**: Addresses the effects and side effect management of radioactive iodine therapy in thyroid cancer treatment, thyroid-stimulating hormone administration, and salivary gland function evaluation. **F**: Covers the usefulness of FDG PET/MRI in tumor diagnosis and staging, its advantages in providing soft tissue contrast and anatomical information, and the need for quantitative analysis. **G**: Covers various topics in nuclear medicine and molecular imaging, including advancements in imaging techniques, radiopharmaceutical development, cancer therapy, stem cell tracking, and theranostics. **H**: Primarily focuses on the role of molecular imaging in genitourinary cancers, particularly the diagnostic use of PSMA PET/CT in prostate cancer and recent insights on targeted radionuclide therapy

Identifying the latest research trends and discovering promising research topics is a critical task for researchers. Utilizing the GPT API can help analyze large volumes of paper abstracts to identify research trends [31]. By retrieving abstracts from the Pubmed or other database, converting them into numerical representations through embedding, and then extracting and clustering important information. Embedding is capturing the semantic meaning and context of words or documents and converting it into a vector that a machine learning model like GPT can understand. This process can use GPT’s embedding model, which maps words or documents to a high-dimensional vector space [32]. And then by presenting clustered abstracts to GPT and asking it to write a representative topic for each cluster, the contours of research trends become apparent. Additionally, it is possible to analyze the main concepts that appear within each cluster using Named Entity Recognition methods. If organized together with publication year information, trend analysis based on years becomes feasible. Figure 3 demonstrates the research trend analysis of the Nuclear Medicine and Molecular Imaging journal from 2010 to 2023 using GPT.

Additionally, similarity search can be utilized to find papers highly related to each topic. For example, searching for the latest trend, “H: Primarily focuses on the role of molecular imaging in genitourinary cancers, particularly the diagnostic use of PSMA PET/CT in prostate cancer and recent insights on targeted radionuclide therapy,” yields relevant papers such as “Current Status of PSMA-Targeted Radioligand Therapy in the Era of Radiopharmaceutical Therapy Acquiring Marketing Authorization” [33] with a similarity score of 0.200, “Technetium 99m PSMA Superscan Mimicking a Bone Scan Gone Wrong” [34] with a similarity score of 0.2210, “The Emergence of Theranostics in the Philippines: Overcoming Challenges and Bringing Hope” [35] with a similarity score of 0.2282, and “¹⁶¹Tb-PSMA Unleashed: a Promising New Player in the Theranostics of Prostate Cancer” [36] with a similarity score of 0.2299.

### IRB and Project Proposal Pre-Assessment

GPT can also assist researchers in the writing and evaluation of IRB and project proposals. By uploading self-inspection checklists and other relevant materials to GPT, researchers can instruct the model to check for specific requirements and criteria. This feature can be repeatedly used throughout the proposal preparation process, saving time and effort. Additionally, by reviewing the self-inspection checklist, researchers can gain insights into the necessary content for their proposals. However, it is crucial to provide GPT with specific and well-defined prompts for each required item to ensure accurate evaluation. For instance, if GPT is asked to assess the presence of the “number of research subjects,” it should be provided with a clear criterion for satisfaction, such as a minimum number of subjects required, to avoid accepting insufficient entries.

When writing IRB and project proposals, more information needs to be provided than just pre-assessment. In addition to the various types of materials mentioned earlier, a clear outline and format must be explicitly presented. Although GPT’s primary output is mainly text, proposals with only text can be dull. While GPT can generate images, its ability to create charts and diagrams is still lacking, making it difficult to use for this purpose. Therefore, it is recommended to use GPT to produce intermediate products for the proposal rather than directly creating the final output. Additionally, while GPT can perform web searches for reference materials, it has limitations in finding information if access is restricted.

## Limitations and Ethical Considerations

While GPT offers numerous benefits for scientific research, it is essential to acknowledge its limitations and consider the ethical implications of its use [37]. One of the main concerns is the potential for hallucination and inaccurate information generation [38]. GPT’s vast knowledge is based on the data it was trained on, and it may sometimes generate information that deviates from the truth. This phenomenon is particularly likely to occur when GPT is asked questions that require the most up-to-date information. To mitigate this issue, researchers should always verify the reliability of the sources and critically review GPT’s responses. Blindly accepting GPT’s output without fact-checking can lead to the propagation of misinformation and compromise the integrity of the research.

It is crucial to emphasize that GPT is a tool to assist researchers, but the researchers themselves should always remain the primary agents of the research. GPT cannot replace human expertise, critical thinking, and ethical judgment. Researchers must take responsibility for the outcomes of their research and ensure that the use of GPT aligns with established research ethics and guidelines. Additionally, it is important to note that each journal has different policies regarding the use of GPT, so researchers must verify and adhere to these policies.

Applying GPT models to nuclear medicine presents additional challenges due to the field’s technical nature. A significant limitation is the model’s inability to incorporate the most recent findings from academic journals in real-time, leading to potentially outdated or incomplete information. This is especially problematic when applying the latest advancements, such as novel PET scan technologies or new radiopharmaceuticals. To address this issue, researchers can use techniques like Retrieval-Augmented Generation to access the most current information from relevant sources in real-time.

Furthermore, biases in the training data can lead to skewed results. For example, the term “bone scan” is often interpreted as an X-ray image of the bones. To avoid confusion, it should be specified as “bone scintigraphy.” Additionally, when responding to treatment recommendations, if guidelines are not explicitly mentioned, the model may primarily answer based on the guidelines it has been trained on, without considering factors such as race, nationality, age, gender, or regional characteristics.

## Conclusion

This review article has explored the potential of GPT in scientific research, highlighting its core functions, key features, and real-world applications. GPT’s ability to understand and generate human-like language, combined with its emergent abilities such as instruction following, in-context learning, and step-by-step reasoning, makes it a powerful tool for researchers. By leveraging GPT’s capabilities, researchers can streamline their workflows, uncover novel insights, and accelerate the pace of scientific discovery.

To fully harness the potential of GPT, researchers must develop an understanding of prompt engineering. Designing clear, specific, and context-aware prompts is essential for eliciting desired outputs from GPT. As the field of prompt engineering evolves and researchers share their experiences and best practices, the collective intelligence surrounding GPT utilization will grow, leading to more effective and efficient applications in scientific research.

However, it is essential to acknowledge its limitations and consider the ethical implications of its use. Researchers must critically evaluate GPT’s outputs and uphold their responsibility as the primary agents of the research process. It is crucial to understand and approach GPT as a tool to augment human expertise rather than replace it.

## Data Availability

Contact the corresponding author for data requests.

## Declarations

### Generative AI in scientific writing

We would like to acknowledge the assistance of language models Claude by Anthropic in the preparation of this manuscript. These AI-based tools were used to aid in the drafting process, however, the authors thoroughly reviewed and edited the generated content to ensure accuracy and take full responsibility for the final manuscript.
