# Correction to: How to Harness the Power of GPT for Scientific Research: A Comprehensive Review of Methodologies, Applications, and Ethical Considerations

**DOI:** 10.1007/s13139-024-00888-9

**Published:** 2024-11-01

**Authors:** Ki-Seong Park, Hongyoon Choi

**Affiliations:** 1 Department of Nuclear Medicine, Chonnam National University Hospital, 42 Jaebong-ro, Dong-gu,, Gwangju , 501-757 Republic of Korea; 2 Department of Nuclear Medicine, Seoul National University Hospital, 101 Daehak-ro, Jongno- Gu, Seoul, 03080 Republic of Korea; 3 Department of Nuclear Medicine, Seoul National University College of Medicine, 101 Daehak-ro, Jongno- Gu, Seoul, 03080 Republic of Korea

**Correction to: Nuclear Medicine and Molecular Imaging (2024) 58:323–331**

10.1007/s13139-024-00876-z

The article How to Harness the Power of GPT for Scientific Research: A Comprehensive Review of Methodologies, Applications, and Ethical Considerations, written by Ki-Seong Park and Hongyoon Choi, was originally published Online First without Open Access. After publication in volume 58, issue 6, pages 323–331, the author decided to opt for Open Choice and to make the article an Open Access publication. Therefore, the copyright of the article has been changed to © The Authors 2024 and the article is forthwith distributed under the terms of the Creative Commons Attribution (CC BY) license 4.0 International License, which permits use, sharing, adaptation, distribution and reproduction in any medium or format, as long as you give appropriate credit to the original author(s) and the source, provide a link to the Creative Commons licence, and indicate if changes were made. The images or other third party material in this article are included in the article’s Creative Commons licence, unless indicated otherwise in a credit line to the material. If material is not included in the article’s Creative Commons licence and your intended use is not permitted by statutory regulation or exceeds the permitted use, you will need to obtain permission directly from the copyright holder. To view a copy of this licence, visit http://creativecommons.org/licenses/by/4.0.

The original article has been corrected.

